# *Plasmodium knowlesi* in Human, Indonesian Borneo

**DOI:** 10.3201/eid1604.091624

**Published:** 2010-04

**Authors:** Melanie Figtree, Rogan Lee, Lisa Bain, Tom Kennedy, Sonia Mackertich, Merrill Urban, Qin Cheng, Bernard J. Hudson

**Affiliations:** Royal North Shore Hospital, Sydney, New South Wales, Australia (M. Figtree, T. Kennedy, S. Mackertich, B.J. Hudson); Westmead Hospital, Sydney (R. Lee); Australian Army Malaria Institute, Enoggera, Queensland, Australia (L. Bain, Q. Cheng); Mona Vale Hospital, Sydney (M. Urban)

**Keywords:** Plasmodium knowlesi, imported malaria, malaria, human, parasites, Indonesian Borneo, dispatch

## Abstract

*Plasmodium knowlesi* is now established as the fifth *Plasmodium* species to cause malaria in humans. We describe a case of *P. knowlesi* infection acquired in Indonesian Borneo that was imported into Australia. Clinicians need to consider this diagnosis in a patient who has acquired malaria in forest areas of Southeast Asia.

*Plasmodium knowlesi* is now recognized as a cause of potentially fatal human malaria in forest areas of Southeast Asia. We describe a case of *P*. *knowlesi* malaria acquired in Indonesia and imported to Australia.

## The Patient

A 39-year-old man from Australia came to a suburban hospital in Sydney, New South Wales, Australia, with a 2-week history of morning fevers and mild headaches. His symptoms started 13 days after he left Indonesian Borneo (Kalimantan). The patient had spent an average of 10 days per month for the past 18 months working adjacent to a forest area in South Kalimantan Province, Indonesian Borneo. The most recent visit was toward the end of the rainy season. He did not use any personal vector avoidance measures (mosquito nets, long clothing, insect repellent) or receive malaria chemoprophylaxis. The patient did not travel to any other malaria-endemic areas during this 18-month period.

He did not have a remarkable medical history. On examination, he was febrile (38.9°C) and had a heart rate of 88 beats/min, blood pressure of 128/78 mm Hg, normal respiration rate, and oxygen saturation of 99% on room air. Physical examination was unremarkable. Laboratory investigations showed mild thrombocytopenia (106 × 10^9^ cells/L, reference range 150–450 × 10^9^ cells/L), mild leukopenia (3.7 × 10^9^ cells/L, reference range 4.3–10 × 10^9^ cells/L), and unremarkable results for levels of hemoglobin (142 g/L, reference range 130–180 g/L), bilirubin (12 μmol/L, reference value <20 μmol/L), and creatinine (95 μmol/L, reference range 40–120 μmol/L).

Malaria parasites were seen on Giemsa-stained thick and thin blood films (parasitemia level 185 parasites/μL). Parasite morphologic features resembled those of *P*. *malariae* with typical trophozoite band forms and heavily pigmented schizonts found inside smaller erythrocytes (Figure). Some parasites had morphologic features similar to those of *P*. *falciparum*. These similarities included ring forms and mature trophozoites with stippling of erythrocytes (Figure, panel B). A rapid diagnostic test result for histidine-rich protein 2 of *P*. *falciparum* was negative.

**Figure:**
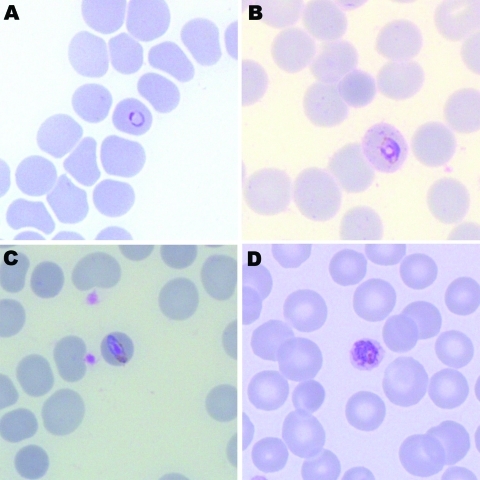
Giemsa-stained thin blood films of patient infected with *Plasmodium knowlesi*, showing a ring form (A), a trophozoite with Sinton and Mulligan stippling (B), a band form resembling *P*. *malariae* (C), and an early schizont (D). Original magnification ×100.

Given increased reports of *P*. *knowlesi* in Malaysian Borneo, we conducted molecular studies to identify the species. Results of multiplex PCRs for *P*. *falciparum*, *P*. *vivax*, *P*. *malariae*, and *P*. *ovale* were negative (*1*). Results of a PCR using *P*. *knowlesi*–specific primers (*2*) were positive for undiluted and diluted (1:50) blood samples. Sequencing of a small subunit rRNA gene product showed 100% identity with *P*. *knowlesi* (National Center for Biotechnology Information accession no. GU049678).

The patient was treated with atovaquone/proguanil, 250 mg/100 mg, 4×/day for 3 days. His fever resolved and the thrombocyte count returned to the reference level within 48 hours. He did not show any complications.

## Conclusions

Naturally acquired human infection with *P*. *knowlesi* was first described in Malaysian Borneo in 1965 after an unusual sequence of events (*3*). Extensive investigation at this time failed to demonstrate zoonotic transmission of simian malaria to humans. More recently, molecular techniques have identified human infections with *P*. *knowlesi*, establishing it as the fifth *Plasmodium* species that infects humans (*2*). *P*. *knowlesi* accounts for most (70%) human malaria infections requiring hospitalization in Sarawak, Malaysian Borneo (*4*), and is widespread throughout Malaysia (*5*). Reports have also described human infections in Thailand (*6*), along the border of the People’s Republic of China and Myanmar (*7*), Singapore (*8*), and the Philippines (*9*). A recent epidemiologic study reported that 4/22 malaria cases diagnosed by microscopy as *P*. *falciparum*, *P*. *vivax*, or mixed *P*. *falciparum*/*P*. *vivax* infections were identified retrospectively by PCR to be mixed infections that included *P*. *knowlesi* (*10*). Presumably, *P*. *knowlesi* may account for a higher proportion of cases if those diagnosed morphologically as *P*. *malariae* were investigated.

Human malaria was considered to be caused by only 4 *Plasmodium* species in the premolecular biology era. Simian and human malaria parasites, including *P*. *knowlesi* and *P*. *malariae*, are often indistinguishable morphologically. Clues to identification of *P*. *knowlesi* by light microscopy that are useful, if present, include early trophozoites with fine ring forms, double chromatin dots, and 2–3 parasites per erythrocyte (resembling *P*. *falciparum*), trophozoites with a bird’s-eye appearance, mature trophozoites with a band appearance resembling *P*. *malariae* (Figure, panel C), and mature schizonts with a higher average merozoite count (16/erythrocyte) than in *P*. *malariae* (12/erythrocyte) (*2**,**11*).

Commercially available rapid diagnostic tests do not distinguish *P*. *knowlesi* from other forms of human malaria parasites. Lactate dehydrogenase produced by the 4 other *Plasmodium* spp. (pLDH) that cause human malaria is also present in *P*. *knowlesi*. Antibodies specific for pLDH of *P*. *falciparum* and *P*. *vivax* cross-react with pLDH of *P*. *knowlesi* (*12*) and therefore cannot be used to reliably distinguish *P*. *knowlesi* from mixed infections.

Distinction of *P*. *knowlesi* from *P*. *malariae* has useful management implications for patients and public health control measures. *P*. *knowlesi* potentially can cause severe disease and death, whereas *P*. *malariae* is generally benign. Daneshvar et al. recently published a prospective study of *P*. *knowlesi* infection in humans (*4*). They reported thrombocytopenia in 100% (107/107) of persons infected with *P*. *knowlesi* and lower mean ± SD thrombocyte counts (71 ± 35 × 10^9^ cells/L) than in persons infected with *P*. *falciparum* (108 ± 59 × 10^9^ cells/L) or *P*. *vivax* (118 ± 51 × 10^9^ cells/L). Mean parasitemia level was 1,387 parasites/μL; 30.8% (33/107) of the case-patients had <500 parasites/μL. Severe infection was found in 7 (6.5%) of 107 patients, and the case-fatality rate was 1.8% (2/107) among hospitalized patients (*4*).

Deaths and severe disease caused by *P*. *knowlesi* result from pulmonary and hepatorenal failure (*5*). Severity of *P*. *knowlesi* infection is related to potentially high parasitemia levels produced by its rapid and unique 24-hour erythrocytic cycle and its ability to infect all stages of erythrocytes (*13*). Sequestration is not thought to occur during *P*. *knowlesi* infection, and neurologic complications seen during *P*. *falciparum* infection have not been described. Although our patient was treated with atovaquone/proguanil, patients with similar uncomplicated cases have responded well to treatment with chloroquine (*4*).

Public health control is challenging in areas where zoonotic human malaria is endemic (*14*). Standard public health measures for malaria prevention (insecticide-treated nets, indoor residual spraying, and intermittent preventive treatment in the reservoir population) are likely to be less effective than for typical forms of human malaria. Nevertheless, travelers to malaria-endemic areas should be encouraged to practice mosquito bite protection measures and chemoprophylaxis.

*P*. *knowlesi* malaria is transmitted from long-tailed (*Macaca fasicularis*) and pig-tailed (*M*. *nemestrina*) macaques to humans by *Anopheles latens* mosquitoes (in the Kapit Division of Malaysian Borneo) when humans visit forest or forest fringe areas. However, transmission does not seem to occur readily in villages (*2**,**15*). Increased recognition of *P*. *knowlesi* indicates that human infection is possible by with other simian malaria parasites (*P*. *cynomolgi* and *P*. *inui*).

We report a patient with *P*. *knowlesi* infection that was acquired in Indonesia and imported to Australia. Fortunately, this patient had a low parasitemia level and mild disease. A high degree of clinical suspicion is likely to increase the number of *P*. *knowlesi* cases diagnosed in patients with malaria acquired in forest areas of Southeast Asia.
